# The Ionic Selectivity of Lysenin Channels in Open and Sub-Conducting States

**DOI:** 10.3390/membranes11110897

**Published:** 2021-11-19

**Authors:** Andrew Bogard, Pangaea W. Finn, Fulton McKinney, Ilinca M. Flacau, Aviana R. Smith, Rosey Whiting, Daniel Fologea

**Affiliations:** 1 Department of Physics, Boise State University, Boise, ID 83725, USA; andybogard@u.boisestate.edu (A.B.); pangaeafinn@u.boisestate.edu (P.W.F.); fultonmckinney@u.boisestate.edu (F.M.); ilinca.flacau2023@gmail.com (I.M.F.); avianasmith@u.boisestate.edu (A.R.S.); roseywhiting@u.boisestate.edu (R.W.); 2 Biomolecular Sciences Graduate Program, Boise State University, Boise, ID 83725, USA

**Keywords:** lysenin, selectivity, membrane voltage, sub-conducting channels

## Abstract

The electrochemical gradients established across cell membranes are paramount for the execution of biological functions. Besides ion channels, other transporters, such as exogenous pore-forming toxins, may present ionic selectivity upon reconstitution in natural and artificial lipid membranes and contribute to the electrochemical gradients. In this context, we utilized electrophysiology approaches to assess the ionic selectivity of the pore-forming toxin lysenin reconstituted in planar bilayer lipid membranes. The membrane voltages were determined from the reversal potentials recorded upon channel exposure to asymmetrical ionic conditions, and the permeability ratios were calculated from the fit with the Goldman–Hodgkin–Katz equation. Our work shows that lysenin channels are ion-selective and the determined permeability coefficients are cation and anion-species dependent. We also exploited the unique property of lysenin channels to transition to a stable sub-conducting state upon exposure to calcium ions and assessed their subsequent change in ionic selectivity. The observed loss of selectivity was implemented in an electrical model describing the dependency of reversal potentials on calcium concentration. In conclusion, our work demonstrates that this pore-forming toxin presents ionic selectivity but this is adjusted by the particular conduction state of the channels.

## 1. Introduction

The biological activity of ion channels is a direct consequence of three common characteristics: high transport rate, regulation, and selectivity [1]. Ionic selectivity is a salient feature of ion channels leading to creating and maintaining electrochemical gradients needed for the correct functionality of cells [2,3,4,5,6], and the modulation of these gradients by physical and chemical stimuli is essential for excitability [1,7,8]. In addition, dynamic resting membrane potentials originating in ionic selectivity are now considered critical for a larger variety of processes such as cell cycle, volume control, cell migration, wound healing, and cell proliferation [9]. Another category of membrane transporters that shares similarities with ion channels are represented by pore-forming toxins (PFTs), which introduce conducting pathways in the host membrane, capable of sustaining high transport rates [10]. Some PFTs may also present regulation by physical and chemical stimuli [11,12,13,14], and selectivity [15,16,17,18,19]. Although the ionic selectivity of PFTs is generally far under that of ion channels, prior investigations provided valuable information on the nature of the selectivity filters and even allowed intentional selectivity modulation by chemical modifications [16]. Such achievements are anticipated not only to improve our understanding of the selectivity mechanisms and physiological relevance but also for developing new drugs, therapeutic strategies, and applications in synthetic biology. In this respect, we focused our work on investigating the ionic selectivity of lysenin channels. Lysenin is an intriguing PFT extracted from the red earthworm *E. fetida*, which inserts very stable channels in membranes containing sphingomyelin [13,17,20,21,22,23,24]. The pore-forming mechanism comprises binding to sphingomyelin and deployment of beta-sheets through the host membrane [25,26]. Lysenin exhibits a high transport rate and is also regulated by voltage and ligands [12,13,14]. Interestingly, the voltage-gating feature requires anionic lipids in the host membrane and is suppressed when neutral lipids are used [13]. However, the gating induced by multivalent ions acting as ligands is preserved in both neutral and charged lipid membranes [14]. Previous investigations on lysenin suggest a weak cation selectivity [15,17], as inferred from measuring the membrane voltage achieved upon creating asymmetrical ionic conditions. However, these are single-point measurements, and no extensive study of the selectivity in various ionic conditions is available. To fill this gap in our knowledge, we employed electrophysiology measurements and expanded the investigations on the selectivity of lysenin channels inserted into planar lipid membranes by considering multiple ions and concentration conditions. These investigations allowed us to provide quantitative analyses of the cation selectivity and show differences between cationic ion species (i.e., Na^+^, K^+^, Cs^+^, and Li^+^). In addition, we also demonstrated that lysenin channels present different permeabilities for anionic species (i.e., Cl^−^ and I^−^). These features, identified for fully-open lysenin channels, resemble the selectivity of ion channels. However, ion channels such as VDAC [27,28,29,30], mechano-sensitive, [31,32,33], sodium [34], and potassium [35,36] may undergo conformational transitions that lead to intermediate, sub-conducting states. Although the physiological relevance of sub-conductance is poorly understood, adjustments of the ionic permeabilities in such sub-conducting states have been reported [27,28,29]. For a better understanding of how intermediate conductance states adjust the transport properties of protein pores, we exploited a unique feature of lysenin channels, which is the attainment of stable sub-conducting states in the presence of divalent ions (i.e., Ca^2+^) [12,14]. In this line, our experimental work demonstrates that the selectivity of lysenin channels to monovalent ions is significantly diminished when they are sub-conducting, which leads to vanishing membrane voltages. The diminished selectivity was assessed by employing a simple electrical model, which in conjunction with the Langmuir isothermal adsorption model describing the divalent ion–channel interaction [12] provided a good match of the experimental data. 

## 2. Materials and Methods

### 2.1. Materials

The bilayer lipid membranes (BLMs) used for all the experiments described in this work were composed of asolectin (Sigma-Aldrich, St. Louis, MO, USA), cholesterol (Sigma-Aldrich), and sphingomyelin (Avanti Polar Lipids, Alabaster, AL, USA). The lipids in powder form were solubilized in n-decane (TCI America, Portland, OR, USA) and mixed at a 10:4:4 molar ratio for a final concentration of 50 mg asolectin/mL mixture. NaCl, KCl, CsCl, LiCl, KI, and CaCl_2_ (ThermoFisher Scientific, Waltham, MA, USA) were dissolved in deionized water and buffered with 20 mM Hepes (ThermoFisher Scientific) for a final pH of 7.2. The used solutions were either starting support electrolyte (50 mM salt concentration) or high-concentration stock solutions for ionic additions (2–3 M final concentration). 

Lysenin was produced and purified in our Biomolecular Research Center core facility [37] through established protocols [37,38,39,40]. Briefly, lysenin cDNA for the full-length protein (accession number D85846) was synthesized and subcloned in pMAL-c5x (GenScript, Piscataway, NJ, USA). This construct generates a maltose-binding protein-linked lysenin with a Factor Xa cleavage site between them. Following transformation of the plasmid into *Escherichia coli* BL21 competent cells and purification [37,38,39,40], the full-length protein was thoroughly tested for activity and transport properties: channel insertion and voltage regulation [13,17], ligand-induced gating [12,14], and attainment of sub-conducting states in the presence of Ca^2+^ ions [12,14]. A stock solution of lysenin dissolved in Phosphate Buffered Saline (PBS1x, pH = 7.2, Sigma-Aldrich) at 1 µg/mL concentration was utilized for channel reconstitution in artificial membranes. All other common chemicals were purchased from various producers and distributors.

### 2.2. Methods

#### 2.2.1. BLM Production, Characterization, and Channel Insertion

To investigate the selectivity of lysenin channels we used a typical planar BLM setup detailed in prior work [12]. The membrane was formed by the painting method in a small hole (~100 µM diameter) produced in a thin PTFE film (125 µm thickness) by an electric spark. The two ~ 1 mL reservoirs were filled with support electrolyte (50 mM salt, if not otherwise indicated). The electrical connections with the Axopatch 200B amplifier (Molecular Devices, San Jose, CA, USA) were made via Ag/AgCl electrodes connected to the electrolyte solutions through salt bridges made with 2% TopVision Low Melting Point Agarose (ThermoFisher Scientific) and 1 M NaCl to minimize the junction potential. The electrodes were wired to the headstage of the electrophysiology amplifier. The signal was digitized with the Digitizer 1440A (Molecular Devices) and recorded on a computer for further analysis. All the experiments were performed at room temperature (22.5 ± 0.5 °C). 

The bilayer formation and its integrity were monitored by employing membrane capacitance and conductance measurements. After a stable and intact, non-conducting membrane was formed, channel insertion was initiated by the addition of small amounts of lysenin (~0.3 nM final concentration) to the grounded reservoir. Channel insertion was observed as a step-wise variation of the ionic currents recorded at −80 mV bias potential (to avoid the voltage-induced gating) and under solution stirring with a low noise magnetic stirrer (Warner Instruments, Hamden, CT, USA). 

#### 2.2.2. Voltage Measurements

**Figure 1:**
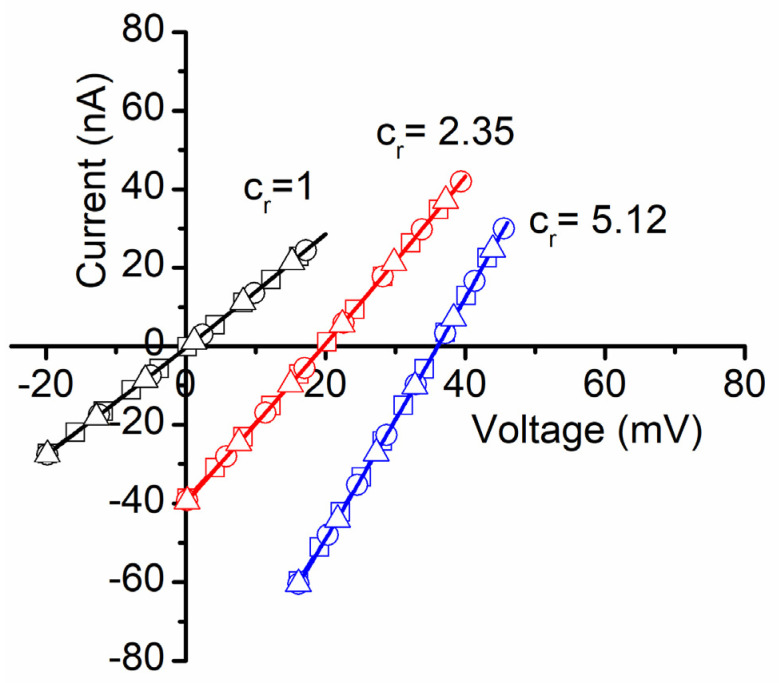
Experimental determination of membrane voltages for lysenin channels reconstituted into planar BLMs. The addition of NaCl to the grounded reservoirs leads to a right shift of the IV plots, indicative of membrane voltage occurrence. An individual experiment employed three IV sweeps for each NaCl concentration ratio c_r_, and their average was used to calculate the membrane voltage as the x-intercept. All the points in the plots are experimental; the symbols have been added as visual aids to discriminate between sweeps recorded in identical experimental conditions.

Insertion completion was indicated by stable ionic currents, achieved in approximately one hour after lysenin addition. The amplitude of the macroscopic ionic currents and the unitary conductance of single channels were used to estimate the number of inserted channels, which varied from several hundred to a few thousand for each membrane we used. After stabilization, we determined the membrane voltages originating from the chemical gradients produced by the addition of small and known amounts of concentrated ionic solutions to the grounded reservoir. The experimental data were recorded with pClamp 10.6.2.2 (Molecular Devices), plotted with Clampfit 10.6.2.2 (Molecular Devices), and the membrane voltages V_m_ determined as the x-intercept of the IV plots (the reversal potentials) recorded for each of the asymmetrical ionic conditions (including symmetrical ionic conditions controls) [7,15,17,18,30]. For each IV plot, we set a voltage range of 20 mV and lower and upper limits such that the plots crossed the x-axis. A single voltage sweep was set for 20 s, and the sampling rate was set to 10 samples/s (hence a 0.1 mV resolution). As detailed in Figure 1 as an example for NaCl, for each of the concentration ratio (c_r_) conditions (c_r_ = 1, 2.35, and 5.12, respectively) we recorded three sweeps within the same experiment; the excellent overlapping of the sweeps for identical ionic conditions indicates attaining a steady state. The linear fit of the experimental data in Clampfit provided the y-intercepts and slopes, which were next used to determine the x-intercepts (membrane voltages). 

The averaged values of the membrane voltage were determined by using a custom-made analysis program in R, which also calculated the concentration ratios for each of the experimental conditions by accounting for added ions. The averaged membrane voltages and concentration ratios were plotted and fitted (Origin 8.5.1, OriginLab Corporation, Northampton, MA, USA) with the Goldman–Hodgkin–Katz (GHK) equation [7,15,16,17,18,30], adjusted for one cation (C^+^) and one anion (A^−^) monovalent ion species:

(1) $$\mathrm{Vm}=-\frac{\mathrm{RT}}{F}\ln\left(\frac{P_{C^{+}}{{[C}^{+}]}_{h}{+P}_{A^{-}}{{[A}^{-}]}_{g}}{P_{C^{+}}{{[C}^{+}]}_{g}{+P}_{A^{-}}{{[A}^{-}]}_{h}}\right)$$

where R is the universal gas constant (R = 8.31 J mol^−1^ K^−1^), T is the absolute temperature, F is the Faraday number (F = 96,485.35 C mol^−1^), P denotes the permeabilities of the ionic species, and the square brackets indicate their concentrations in the ground (g) and headstage (h) reservoirs, respectively. 

The GHK fit provided the permeability ratio of cations to anions (i.e., P_C+_/P_A−_) under the assumption that ionic diffusion does not change the ionic concentrations achieved by salt addition, i.e., the concentrations of cations and anions in the same reservoir are equal after electrochemical equilibrium is achieved. This assumption is fully justified by the very small number of ions (relative to the bulk number) needed to diffuse for establishing the membrane voltages. The experiments for determining the membrane voltages and estimating the permeability ratios were independently replicated three times, and the statistical analyses performed in Origin 8.5.1 provided the average values and standard deviations of the permeability ratios. No significant differences in membrane voltages were determined from experiments comprising identical solution conditions but different numbers of inserted channels. 

#### 2.2.3. Investigations on Sub-Conducting Channels

To investigate the selectivity of lysenin channels in sub-conducting conditions we utilized buffered KCl (50 mM) solutions as starting support electrolytes. The first set of experiments comprised sub-conductance achievement by the addition of CaCl_2_ to both reservoirs (36 mM final concentration), followed by measurements of the membrane voltage upon successive KCl additions to the grounded reservoir. The next set of experiments comprised achieving a membrane voltage by establishing chemical gradients with KCl addition to the grounded reservoir, followed by monitoring the changes in the membrane voltage upon additions of CaCl_2_ to both reservoirs. The membrane voltage was determined from the IV plots by following the same procedure described above for monovalent ions. The influence of the Ca^2+^ ions on permeability was assessed by employing an electrical model of the membrane and equations detailed in the corresponding results and discussion section. 

## 3. Results

### 3.1. Lysenin Channels in the Open State Are Cation-Selective 

**Figure 2:**
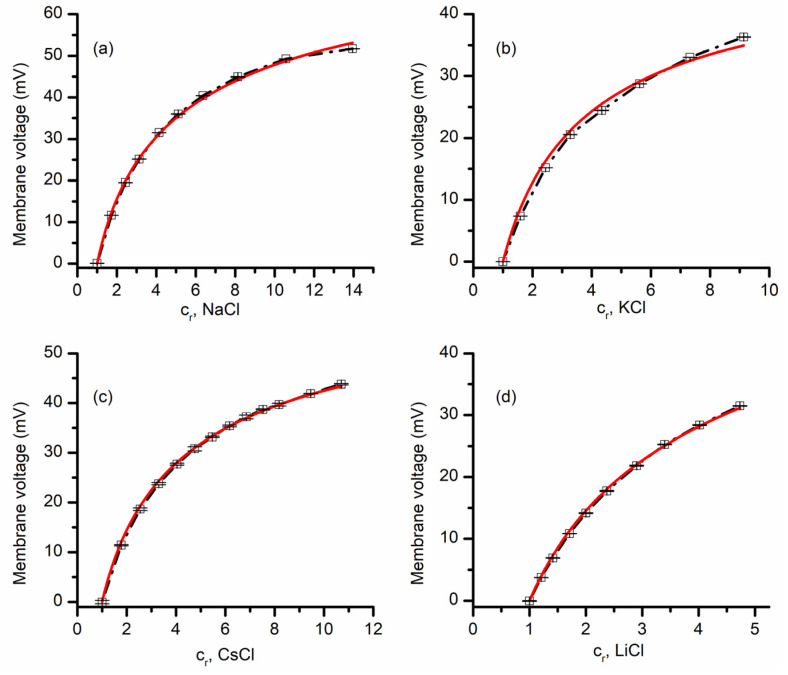
Lysenin has selectivity for monovalent cations. The membrane voltage as a function of concentration ratio c_r_, measured for NaCl (**a**), KCl (**b**), CsCl (**c**), and LiCl (**d**). Each experimental point in the plot (symbols) shows the average value ±SD (n = 3) from three sweeps recorded in single experiments. The full lines are the fit with the GHK equation (single experiments), which was used to determine the permeability ratio of cations over anions from the membrane voltage and concentration ratio c_r_ values for each of the indicated ionic species.

The selectivity of lysenin channels for monovalent ions was first investigated for chlorides by additions of small volumes (ranging from 10 to 50 uL) of buffered 3M stock solutions to the grounded reservoir. The starting solutions in both reservoirs were buffered 50 mM electrolyte solutions of the same chemical species as stocks, except for experiments that comprised mixtures of chlorides and iodides (detailed in the Results section). The control IV plots were run to ensure the absence of any offset transmembrane voltage in symmetrical ionic conditions, after which the membrane voltages were determined for each particular chemical gradient. The results depicted in Figure 2 shows typical recordings (i.e., average values obtained from three sweeps within the same experimental setup) and they fit with the GHK equations for NaCl, KCl, CsCl, and KCl. Subsequent addition of chlorides to the grounded reservoir led to the development of transmembrane voltages with an amplitude that increased monotonically with the concentration ratios (Figure 2) for each of the ionic species, which is a direct consequence of ionic selectivity. The P_C+_/P_A−_ values determined from three independent experiments for the different ionic compositions are 22.4 ± 1.3 for NaCl, 7.5 ± 0.5 for KCl, 12.3 ± 0.5 for CsCl, and 12.9 ± 0.9 for LiCl. All these values clearly indicate that lysenin channels are slightly selective for cations, as suggested in prior work from single-point measurements [15,17]. As we have no reason to assume that the permeability of Cl^−^ is different when the ions originated in the dissociation of different salts, the different permeability ratio values also indicate that the selectivity of lysenin channels also manifests between cationic species. For example, our experimental results suggest that the permeability for Na^+^ is ~3 times larger than the one for K^+^, while Cs^+^ and Li^+^ have similar permeabilities. Such features are essential for many ion channels; however, their capabilities with regard to discriminating between similar ionic species may attain significantly larger values compared to lysenin channels. 

### 3.2. Lysenin Channels in an Open State Present Different Permeability for Anions 

**Figure 3:**
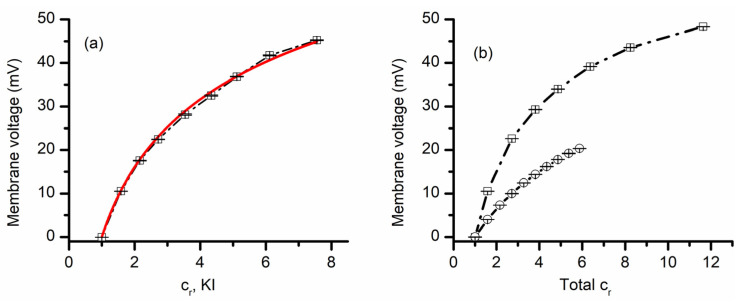
Lysenin channels present different permeability for anions. The membrane voltage as a function of concentration ratio measured for KI addition to a KI support electrolyte solution (**a**). The full line is the fit with the GHK equation, which was used to determine the permeability ratio of K^+^ over I^−^. Panel (**b**) shows the measured membrane voltage as a function of total salt concentration ratio when KI was added over 50 mM KCl (open circles), or KCl was added over 50 mM KI (open squares) to the grounded reservoir. The dashed line is used as a visual aid. For each panel, the experimental points in the plot (symbols) show the average value ± SD (n = 3) from three sweeps recorded in individual experiments.

The above experiments provided information with regards to lysenin channels’ selectivity for cations versus anions, together with an indication that lysenin also discriminates between similar cation species. Next, we asked whether lysenin channels present different selectivity for similar anion species. To answer this question, we ran experiments employing KI for the 50 mM support electrolyte and a 3 M KI stock solution for creating chemical gradients. The plot of the transmembrane voltage versus concentration ratio depicted in Figure 3 shows that a concentration ratio of ~5 led to a transmembrane voltage of ~32 mV. This value is slightly larger than what was obtained for a similar KCl ratio (see Figure 2), indicative of different permeabilities of I^−^ and Cl^−^ ions. The permeability ratio P_C+_/P_A−_ determined from the GHK equation for KI (i.e., 27.3 ± 0.7) was larger than what we obtained for the KCl case (~7.5); this result suggests that the lysenin’s permeability for I^−^ is a few times smaller than for Cl^−^. Since the two permeabilities are different, we predicted that the membrane voltages established across a membrane containing lysenin channels would be different from the addition of KI over KCl support solutions, or KCl over KI. This prediction was confirmed experimentally (Figure 3). The addition of KCl over KI support electrolyte produced a membrane voltage significantly smaller than what was measured for the same concentration ratios when KI was added over KCl. Moreover, the addition of KCl over KI resembled the KCl-only behavior (shown in Figure 2), while KI addition over KCl was rather similar to the KI-only measurements (Figure 3a).

### 3.3. Investigations on the Selectivity of Lysenin Channels in Sub-Conducting States 

**Figure 4:**
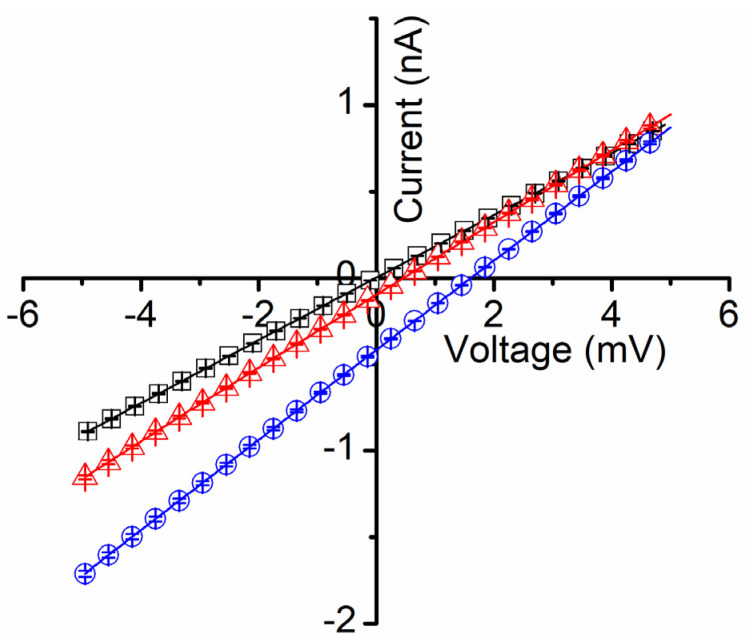
Lysenin’s selectivity is diminished in sub-conducting states. After exposure of lysenin channels to 36 mM Ca^2+^ ions (for inducing stable sub-conducting states), symmetrical KCl concentration conditions indicated the absence of any membrane voltage (open squares). KCl addition to the ground side (concentration ratio of 3 (open triangles), and 13 (open circles), respectively) led to the development of negligible membrane voltages, in the mV range. All the data in the plots are experimental; the symbols have been added as a visual aid, and they represent average values ± SD from three IV sweeps recorded in the same experiments.

Our next investigations focused on less explored aspects of gated channels, which are potential hidden changes of their biophysical properties that occur when the conformational changes lead to transitions to sub-conducting states. Several channels are known to undergo sub-conducting states [29,31], i.e., an intermediate state between open and closed, characterized by a non-zero, intermediate conductance. The physiological relevance of such sub-conducting states is still unclear [28], and deep investigations of such aspects are hindered not only by the limited channel species that present such states but also by a certain inability to control these generally unstable states. Lysenin is a notable exception, and prior experiments demonstrate that lysenin channels may be forced to adopt a stable sub-conducting state upon exposure to divalent organic and inorganic divalent cations [12]. For example, Ca^2+^ or Mg^2+^ at concentrations over 20 mM force the lysenin channels to adopt a stable half-closed state, for which the conductance is ~20% of the conductance of a fully open channel [12]. Therefore, we exploited this remarkable property of lysenin channels to investigate their selectivity in sub-conducting states and compare it with the open state. To achieve this objective, we first tested the development of a transmembrane voltage by imposing non-symmetrical ionic concentrations of KCl upon a membrane containing sub-conducting lysenin channels. In this respect, we used a typical experimental approach for inserting fully open lysenin channels in a membrane bathed by 50 mM KCl. After stabilization, we added 36 mM CaCl_2_ to both reservoirs to force the channels to transition to a sub-conducting state. Although prior work suggests that Ca^2+^ ions permeate the lysenin channels in both open and sub-conducting states [12], we added CaCl_2_ to both sides to avoid asymmetrical divalent ion concentrations potentially leading to supplementary membrane voltages; the absence of such a voltage was verified by running an IV plot from −10 mV to 10 mV, which showed the absence of any ionic current at 0 mV (Figure 4). Next, we exposed the grounded side of the membrane to increased concentrations of KCl by adding to it small amounts of the 3M KCl solution. To our surprise, none of the additions revealed a transmembrane voltage larger than a few mV (Figure 4) even for concentrations for which we otherwise measured tens of mV in the absence of Ca^2+^ (as shown in Figure 2). 

**Figure 5:**
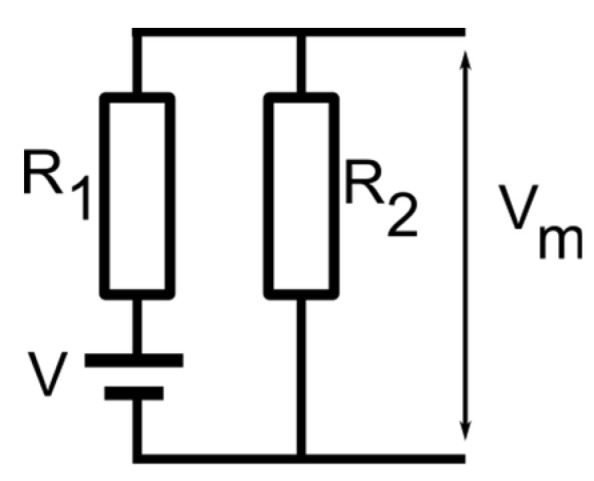
The electrical model for a membrane containing lysenin channels in both open and sub-conducting states. The model includes a voltage source V, open channels of total resistance R_1_, and sub-conducting channels of total resistance R_2_. The resulting membrane voltage V_m_ may be estimated from the electrical parameters of the equivalent circuit.

A reasonable assumption for this unexpected behavior would be the loss of selectivity for channels in the sub-conducting state. We excluded the hypothesis that no ions may move through sub-conducting channels since the ionic conductance in the sub-conducting state is still very large. To reasonably explain the diminished membrane voltage observed experimentally, we modeled the membrane containing lysenin channels in either state by a combination of a voltage source (fully open channels), a serial resistance (R_1_, the resistance of the fully open channels), and another resistance (R_2_) parallel to the entire assembly and representing all the sub-conducting channels (Figure 5). For slow-varying voltage measurements, the capacitance of the membrane may be neglected. Also, although the channels may present a very weak selectivity in the sub-conducting state, we opted to exclude a second voltage source for these channels since the results presented in Figure 4 shows only a negligible voltage for sub-conducting channels exposed to asymmetrical KCl conditions. This low resting voltage also suggests that CaCl_2_ addition, although changes the Cl^−^ concentrations on both sides of the membrane, has at most a small contribution to the membrane voltage. Therefore, the monovalent ions may be considered the major contributors to the membrane voltage for these experimental conditions, which simplifies the electrical model of the membrane.

The membrane voltage for the model shown in Figure 5 may be calculated from Ohm’s law:

(2) $$V_{m}=V\frac{R_{2}}{R_{1}{+R}_{2}}$$

The other premises of the proposed model are as follows: (i) the membrane contains N channels, from which n_1_ are in the open state and n_2_ in the sub-conducting state (N = n_1_ + n_2_); (ii) for given ionic conditions the resistance of an individual open channel is r_1_, and the resistance of an individual sub-conducting channel is r_2_, and (iii) the ratio between the individual resistances of sub-conducting and conducting channels in otherwise identical solution conditions is r_1_/r_2_ = f = 0.2 [12]. With these assumptions, the membrane voltage becomes:

(3) $$V_{m}=\frac{V}{\left(\frac{{\mathrm{fn}}_{2}}{N-n_{2}}\right)+1}$$

Assessing the evolution of the membrane voltage when variable populations of fully conducting and sub-conducting channels coexist in the membrane requires estimating n_2_. This was previously performed by considering the interactions between Ca^2+^ ions and lysenin channels a Langmuir isothermal adsorption process [12], for which:

(4) $$n_{2}=\frac{{K[\mathrm{Ca}}^{2+}]N}{{1+K[\mathrm{Ca}}^{2+}]}$$

where K is the Langmuir constant, [Ca^2+^] is Ca^2+^ concentration in the bulk solution, and N is the total number of lysenin channels inserted into the membrane.

The combination of the last two equations leads to a simplified formula for the membrane voltage:

(5) $$V_{m}=\frac{V}{{\mathrm{fK}[\mathrm{Ca}}^{2+}]+1}$$

**Figure 6:**
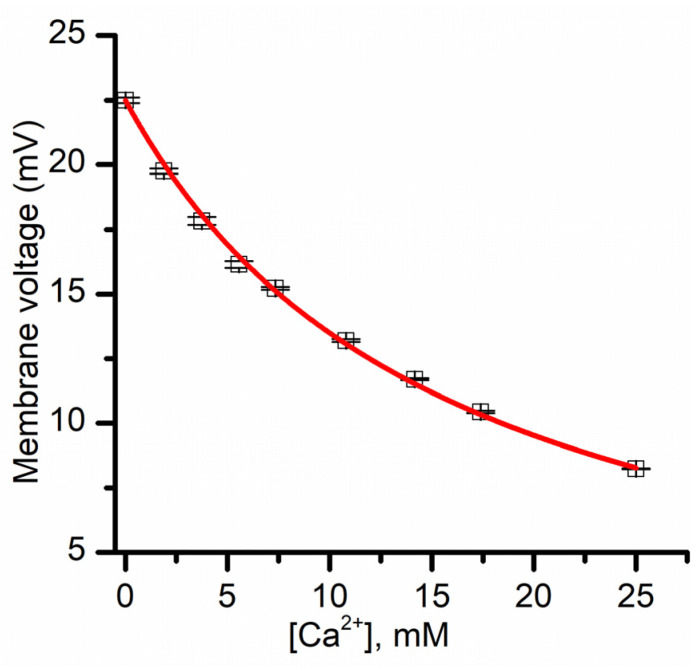
Influence of Ca^2+^ ions on the transmembrane voltage resulting from asymmetrical KCl salt conditions. The membrane voltage (averaged from three sweeps) is diminished upon Ca^2+^ addition to both sides of the membrane, suggesting that lysenin channels in sub-conducting states have reduced selectivity. The continuous line is the fit with Equation (5).

The last equation predicts that the membrane voltage established from chemical gradients must monotonically decrease with increasing Ca^2+^ concentration. To verify this hypothesis, we measured the variation of the membrane voltage created by KCl gradients upon successive Ca^2+^ addition. We utilized buffered KCl (50 mM) support electrolyte and created a membrane voltage of ~22.4 mV by adding KCl to the grounded reservoir (for a concentration ratio of 4.1). The addition of Ca^2+^ up to 25 mM to both reservoirs decreased the membrane voltage in a concentration-dependent manner, as predicted by Equation (5) (Figure 6). The plot was fit with Equation (5), in which we utilized an f value of 0.2, as previously determined from conductance measurements [12]. The fit provided a K value of 0.32 ± 0.07 mM (n = 2). This Langmuir constant is several times larger than what was determined from macroscopic conductance measurements (i.e., K = 0.05 mM [12]). However, those experiments were performed by utilizing 150 mM salt for the supporting electrolyte, and our experiments employed 50 mM. As the primary interaction between divalent metal cations and lysenin channels is presumably electrostatic [12], one may easily anticipate that a lower ionic strength of the solution environment augments the electrostatic interactions by reducing ionic screening; as our experimental determinations indicate, this clearly leads to an increased adsorption constant. 

Ionic selectivity is a salient feature of ion channels, largely utilized for establishing fundamental physiological processes in all living systems. Our work demonstrates that lysenin channels also present ionic selectivity and that the permeability is different for monovalent anions and cations. Although the permeability ratios are much smaller than what is typically encountered for many ion channels, the selectivity function may lead to the development of large resting potentials, comparable to the ones measured for cells. Consequently, this may be further exploited to modulate the membrane voltage of natural membrane systems. Lysenin reconstitution into artificial membrane systems (i.e., liposomes) may provide the means to create electrochemical gradients to be utilized for energy production and control of metabolic processes in synthetic cell-like systems. The ability of lysenin to attain stable sub-conducting states allowed us to investigate its selectivity to monovalent ions, which was significantly diminished. To explain the loss of this function, one may consider it an effect originating in the binding of Ca^2+^ ions to the channel, which will change the electrostatic properties and the energy landscape. However, this may require direct binding to the selectivity filter to annihilate its function. Another hypothesis, which we consider more attractive, is that the channel's conformational changes during the transition from the open to sub-conducting state [12] lead to a displacement of the selectivity filter, hence hindering its function.

The selectivity function presented by lysenin, together with the high transport rate and exquisite regulation by voltage and ligands, closely resembles the salient features of ion channels. It is not clear why a pore-forming toxin meant to kill the target cells is endowed with such remarkable functionalities, which are rather descriptive of ion channels. In this respect, the hypothesis that lysenin is part of a defense system is not fully supported by its unusual characteristics. Future extended work is needed to better understand the ionic permeability of lysenin channels in open and sub-conducting states, identify conformational changes induced by divalent ions, elucidate their physiological role, and develop applications emerging from its unique biophysical properties.
